# Soft-Computing-Based Estimation of a Static Load for an Overhead Crane

**DOI:** 10.3390/s23135842

**Published:** 2023-06-23

**Authors:** Tom Kusznir, Jaroslaw Smoczek

**Affiliations:** Department of Manufacturing Systems, Faculty of Mechanical Engineering and Robotics, AGH University of Science and Technology, al. Mickiewicza 30, 30-059 Kraków, Poland; tkusznir@agh.edu.pl

**Keywords:** overhead crane, payload estimation, identification, genetic programming, sparse regression, subtractive fuzzy clustering

## Abstract

Payload weight detection plays an important role in condition monitoring and automation of cranes. Crane cells and scales are commonly used in industrial practice; however, when their installation to the hoisting equipment is not possible or costly, an alternative solution is to derive information about the load weight indirectly from other sensors. In this paper, a static payload weight is estimated based on the local strain of a crane’s girder and the current position of the trolley. Soft-computing-based techniques are used to derive a nonlinear input–output relationship between the measured signals and the estimated payload mass. Data-driven identification is performed using a novel variant of genetic programming named grammar-guided genetic programming with sparse regression, multi-gene genetic programming, and subtractive fuzzy clustering method combined with the least squares algorithm on experimental data obtained from a laboratory overhead crane. A comparative analysis of the methods showed that multi-gene genetic programming and grammar-guided genetic programming with sparse regression performed similarly in terms of accuracy and both performed better than subtractive fuzzy clustering. The novel approach was able to find a more parsimonious model with its direct implantation having a lower execution time.

## 1. Introduction

Cranes are material-handling machines widely used in the industrial, construction, and logistic sectors. Monitoring the static and dynamic load of material handling systems is important to satisfy safety, reliability, performance, and cost requirements [1,2,3,4]. The weight of the payload is crucial input information in crane applications, such as control, condition monitoring, maintenance planning, prediction of failures, and remaining useful life of crane equipment. Crane cells and scales are commonly used in industrial practice and implemented in different forms in load monitoring systems, such as Load Moment Indicator (LMI) and Rated Capacity Indicator (RCI) [1,2]. For hydraulic cranes, the payload weight is usually determined based on the hydraulic pressures in the lift cylinders [5]. When the load transportation is performed by cranes using ropes, cables, or chains, it requires an additional installation, usually of a strain-gauge-based sensor, onto the hoisting equipment.

The development of controllers for the safe and efficient operation of cranes in terms of crane positioning and payload sway suppression is an active area of research. There have been several control strategies [6] such as partial feedback linearization [7], sliding mode control [8], and nonlinear model predictive control [9] that require information about the operating point of the crane such as payload mass. Payload weight detection also plays an important role in condition monitoring [1,2]. However, in industrial practice there are a variety of crane configurations, thus, when the crane scale installation to the hoisting equipment is not possible, difficult, and costly, deteriorates crane maneuverability, or when the complementary measurement devices for hybrid payload monitoring are required, an alternative solution is to derive information about the load weight indirectly from other sensors. The problem of load estimation for material handling lifting systems in several papers is studied through analytical modeling of their static and/or dynamics. A review of model-based methods of payload estimations in hydraulic excavators and a discussion of their applicability is provided in [10]. The model-based static and dynamic load estimation for hydraulically actuated material handling machines is commonly performed based on information obtained from the hydraulic pressure (pressure difference across the boom) and angular position sensors, while the model parameters are estimated offline and/or online usually using least square [11] or recursive least square method [12]. A variety of models addressing the dynamics of a hoisted load are developed in the literature mostly using the Lagrangian approach, such as a dynamic model of the hoisting motion of a hydraulic lattice mobile crane [13] and an overhead crane hoist with a crane’s structure dynamic model [14,15,16]. An overhead crane hoist with an asynchronous electric motor model is developed and analyzed in numerical simulations to determine the relationship between the weight of a payload and the deviation in the amplitude of the electric motor current [17]. A digital-twin approach with a CAD model of a knuckle boom crane was proposed for real-time monitoring of a payload weight in [18]. A virtual strain gauge sensor was implemented with a nonlinear finite element model of a crane to find a linear relationship between the payload weight input and strain output signals, then the load weight is estimated through the inverse modeling approach applying the input signal from the physical strain gauge used in the experimental setup. The idea to estimate a payload weight using data-driven models identified using machine learning techniques based on data collected from available sensors is implemented in several recent papers. The ANN model trained using the Levenberg–Marquardt algorithm is developed in [19] for payload weight detection of the John Deere 644J hydraulic four-wheel-drive loader based on the differential pressure across the boom cylinder, boom and bucket strokes, and vibration acceleration of the frame. The ANN model is used to estimate a payload mass for a hydraulic forestry crane based on the hydraulic pressure of the inner boom cylinder and the grapple position [20,21]. The vision system and convolutional neural network are reported to recognize a wood log weight for a forestry crane [22]. The quay crane load identification method based on the signals from an accelerometer and strain gauge sensors installed on the crane’s structure parts is reported in [23].

Strain gauge sensors are used in a variety of crane applications. Strain-gauge-based methods are applied to fatigue life prediction of existing crane runway girders and to evaluate the residual operating life of a bridge crane girder [24,25,26]. A combination of strain-gauge measurement and finite element analysis is used to identify crane wheel load for a quay ship-to-shore crane [27]. Strain sensing is used to identify the occurrence and intensity of the overhead crane skewing [28] and in the new technique of payload sway angle measurement [29].

In this paper, a static payload weight is estimated based on the local strain of a crane’s girder and the current displacement of the trolley. The problem of the crane girder’s deformation due to a moving trolley with a rope-suspended payload is studied in the literature mostly using analytical models developed based on the Euler-Bernoulli or Timoshenko beam deformation theories [30,31,32,33,34], or through applying finite elements methods [35,36,37,38]. The analytical models can be used to solve a nonlinear static inverse problem: knowing the deformation of the crane’s girder and trolley displacement, determine the payload mass. However, obtaining an accurate mathematical model can be a challenging process and there are many factors, complexity characteristics, nonlinearities, parameters’ uncertainty, etc., affecting the deviation between a mathematical model and the physical system. Thus, in this paper data-driven techniques are adapted to model-based estimation of a static payload weight. This work follows the previous paper [39], in which a payload weight is estimated for an overhead crane based on a local strain of a girder and the trolley position using a data-driven Takagi–Sugeno fuzzy (TSF) model identified through the subtractive clustering method and least square algorithm, thus, the nonlinear relation between the payload weight, strain, and trolley’s position is approximated by linear models expressed in fuzzy rule consequents. In comparison to the previous work, this paper addresses the issue of improving estimation accuracy and increasing interpretability using genetic-programming-based model structure optimization. Genetic programming is an evolutionary-based method whose outputs are computer programs. This makes genetic programming well suited to find a mathematical function that relates the input to the output, which is interpretable by the user and can be directly implemented reducing execution time. The reduction in execution time of the model is a significant advantage since the payload estimation is carried out online. Two variants of genetic programming, multi-gene genetic programming (MGGP) and grammar-guided genetic programming with sparse regression (G3PSR) are applied to obtain a static data-driven input–output model. The G3PSR algorithm uses sparse regression on a fixed set of evolved candidate model terms that are obtained from a biased search of the function space and the *l*_0_ penalty is added for model selection, which results in reduced complexity and decreases the probability of overfitting. The subsequent sparse regression problem is solved by using the monotonically accelerated proximal gradient descent algorithm [40]. To the best of the authors’ knowledge, the only other evolutionary sparse regression algorithm is based on an elastic net regularization [41]. The contribution of this paper can be summarized as follows:

- Develop a novel genetic programming variant called G3PSR that can be used for symbolic regression problems that can be expressed as a linear in the parameters model.
- Apply genetic programming variants, namely G3PSR and MGGP, to identify a mathematical relationship between the payload mass and the trolley position and girder strain.
- Compare the genetic programming models for mass estimation with a method proposed in the literature [39].

The static model identification process was based on the experimental data obtained from a laboratory overhead crane. The comparative analysis of G3PSR, MGGP, and TSF models is presented in terms of accuracy and complexity. To the best of our knowledge, this is the first work on the crane’s payload weight estimation using a data-driven model identified using multi-gene genetic programming and grammar-guided genetic programming with sparse regression. The other genetic programming-based crane modeling approaches used the MGGP for payload sway prediction [42,43]. Crane dynamics modeling and control were also studied by using a genetic algorithm (GA) in [44,45,46,47].

The rest of the paper is organized as follows. In Section 2, the methods used to identify the models for payload weight estimation are described. Section 3 presents the experimental setup with a laboratory overhead crane, discusses the results of identification experiments, and compares the model performances, while the final conclusions are delivered in Section 4. The lists of symbols and abbreviations used in the paper with their definitions are exposed in Appendix A.

## 2. Methodology

Genetic programming is an evolutionary algorithm that is able to synthesize programs and was developed as an offshoot of genetic algorithms in the 1990s by Koza [48]. One of the important applications of genetic programming has been symbolic regression in which the aim is to identify a mathematical expression that best fits an input–output dataset. In traditional genetic programming, an individual is represented by an abstract syntax tree (AST) whose offspring, obtained by undergoing a variation operation, passes on to the next generation. Different variants of genetic programming have developed, such as multi-dimensional genetic programming, where an individual is composed of different subprograms such as in multi-gene genetic programming [49], linear genetic programming [50], grammatical evolution [51], Keizen programming [52], etc.

### 2.1. Multi-Gene Genetic Programming

Multi-gene genetic programming is a robust variant of genetic programming that was developed in [49]. In MGGP, each individual in the population is composed of a linear combination of functions called genes

(1) $$\hat{y}=\sum_{i=1}^{N}\theta_{i}G_{i}$$

where $\hat{y}$ is the model output, $\theta_{i}$ is the coefficient of the i-th, gene and $G_{i}$ is the result of evaluating the function represented by the *i*-th gene. The expression in (1) allows MGGP to combine traditional genetic programming’s ability to evolve a sequence of functions together with the linear least squares algorithm to improve the performance in terms of speed of finding a solution to the symbolic regression problem as well as accuracy.

**Figure 1:**
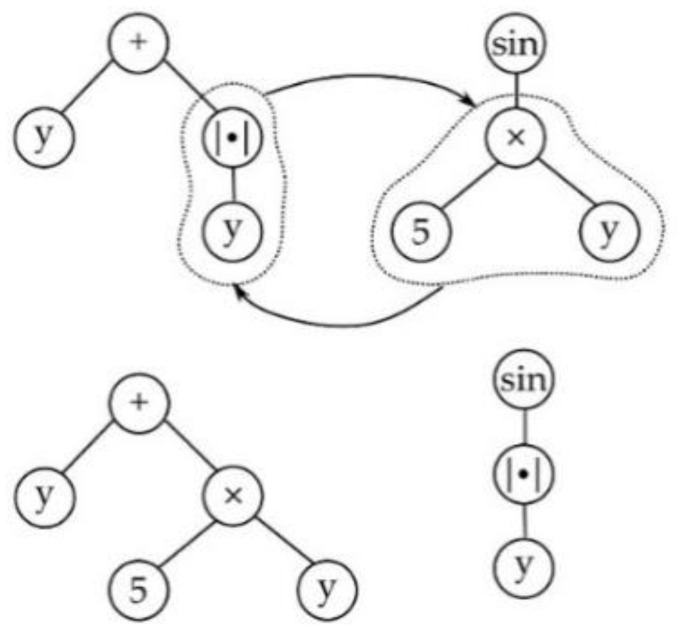
Subtree crossover.

**Figure 2:**
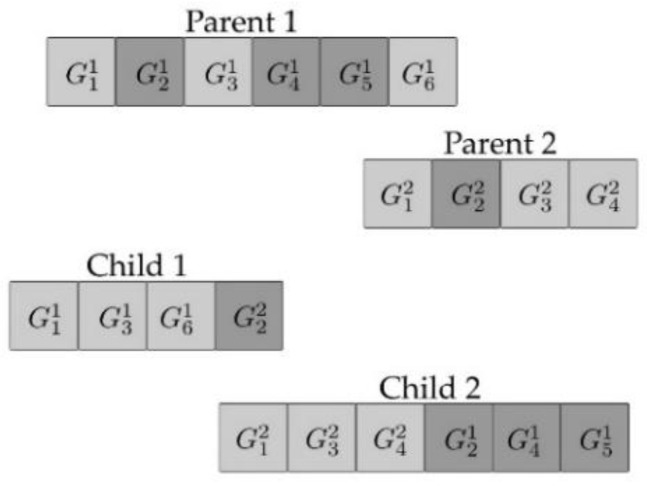
High level crossover.

**Figure 3:**
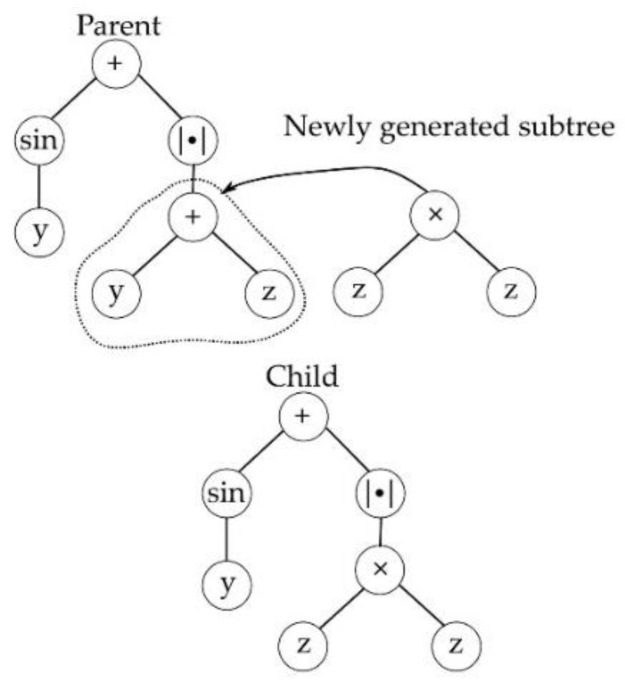
Subtree mutation.

**Figure 4:**
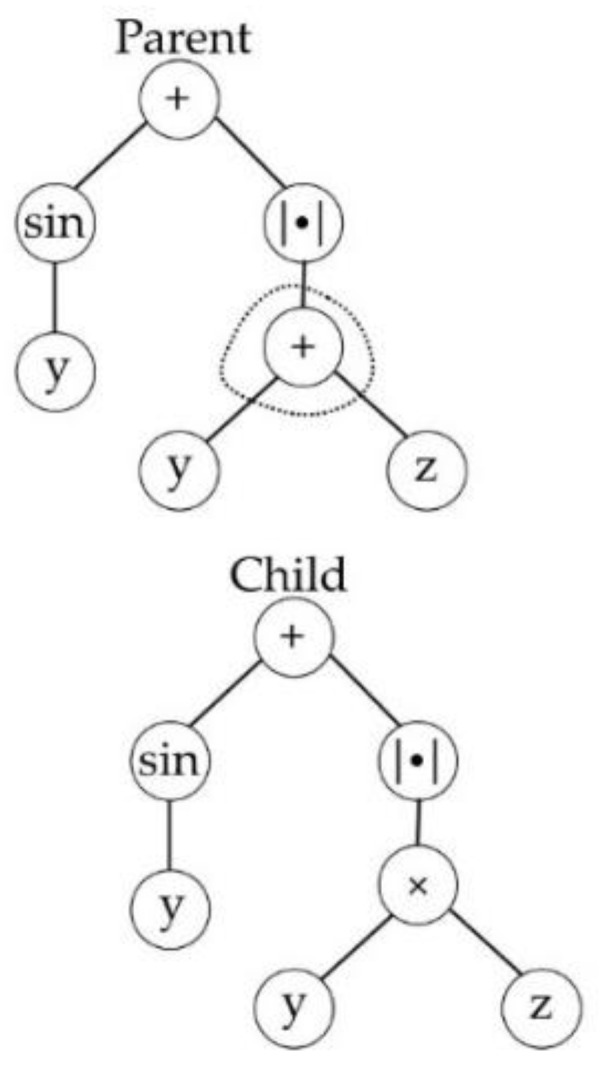
Point mutation.

Selection in MGGP occurs in two stages, at first a parent is selected based on the individual’s fitness, then a gene within the parent is selected at random. Once the gene is selected, it undergoes one of four variation operators: subtree crossover, high-level crossover [53], subtree mutation, and point mutation as shown in Figure 1, Figure 2, Figure 3 and Figure 4. GPTIPS2 [53] an MGGP toolbox for MATLAB is used to obtain the model.

The configuration of the multi-gene genetic programming hyperparameters used to obtain the mathematical expression of the payload mass, m, given the input data x (trolley position) and strain ε were obtained using trial and error based on previously published values [54], with the exception of the maximum number of genes, which was chosen to be 25 in order to have a fair comparison between MGGP and G3PSR, is given in Table 1.

### 2.2. Grammar-Guided Genetic Programming with Sparse Regression

Grammar-guided genetic programming with sparse regression is a novel algorithm in which an individual is composed of $N_{max}$ functions, called model terms, and are the equivalent of genes in MGGP. The difference in representation between MGGP and G3PSR is that in MGGP an individual can have between 1—$N_{max}$ genes, while in G3PSR an individual always has $N_{max}$ candidate model terms. In order to select which model terms are included in the model an $l_{0}$-penalty is added to the regression problem (2) which leads to a sparse solution vector

(2) $$F\left(\theta \right)=\frac{1}{2}{∥y-\phi \theta ∥}_{2}^{2}+\lambda {∥\theta ∥}_{0}$$

where the ${∥x∥}_{0}$ counts the number of nonzero elements in a vector, i.e., ${∥x∥}_{0}=\#\left\{i\;s.t.\;x_{i}\neq 0\right\}$, y is the measured output data, ϕ is a matrix whose columns, *j*, are composed of evaluating the functions represented by the model terms and normalized by $\max\left|\phi_{j}\right|$, θ are the model term coefficients and λ is a constant that promotes the sparsification of the solution. The addition of the $l_{0}$-penalty, which is nonconvex, makes solving (2) NP hard [55]. The solution to (2) is found using the monotone approximated gradient descent algorithm (mAPG) [40]. The pseudocode for mAPG is given in Algorithm 1, where the proximal operator for the $l_{0}$-penalty is the hard thresholding operator (3). The algorithm runs until the maximum number of iterations has been reached or the objective function converges, i.e., $F\left(\theta_{k+1}\right)-F\left(\theta_{k}\right)$ is within a specified tolerance.
**Algorithm 1:** mAPG.**Input:**   ϕ,y,λ **Initialize:** $\rho <1,\;\delta ,\;z_{1}=\theta_{1}=\theta_{0},\;t_{1}=1,\;t_{0}=0,\;k=1$
     **while** not converged **do**         k←k+1         $w_{k}=\theta_{k}+\frac{t_{k-1\;}}{t_{k}}\left(z_{k}-\theta_{k}\right)+\frac{t_{k-1}-1}{t_{k}}\left(\theta_{k}-\theta_{k-1}\right)$         Initialize step size $\eta_{w}$ and $\eta_{\theta }$ using Barzilai-Borwein method      **while** $F\left(z_{k+1}\right)\geq F\left(w_{k}\right)-\delta {∥z_{k+1}-w_{k}∥}_{2}^{2}$
**do**         $z_{k+1}={\mathrm{prox}}_{\eta_{w}\lambda }\left(z_{k}-\eta_{w}\phi^{T}\left(\phi z_{k}-y\right)\right)$         $\eta_{w}=\rho \eta_{w}$
     **end while**
     **while** $F\left(v_{k+1}\right)\geq F\left(\theta_{k}\right)-\delta {∥v_{k+1}-\theta_{k}∥}_{2}^{2}$
**do**         $v_{k+1}={\mathrm{prox}}_{\eta_{\theta }\lambda }\left(v_{k}-\eta_{\theta }\phi^{T}\left(\phi v_{k}-y\right)\right)$         $\eta_{\theta }=\rho \eta_{\theta }$      **end while**
        $t_{k+1}=\frac{\sqrt{4t_{k}^{2}+1}+1}{2}$         $\theta_{k+1}=\{\begin{array}{c} z_{k+1}\\ v_{k+1} \end{array}\begin{array}{l} \mathrm{if}\;F\left(z_{k+1}\right)\leq F\left(v_{k+1}\right)\\ \mathrm{otherwise} \end{array}$
   **end while** **Output:**
θ

(3) $${\mathrm{prox}}_{\gamma {∥v∥}_{0}}=\{\begin{array}{c} \begin{array}{cc} 0 & \left|v\right|\leq \sqrt{2\gamma v} \end{array}\\ \begin{array}{cc} \upsilon & \left|v\right|>\sqrt{2\gamma v} \end{array} \end{array}$$

The variation operators used in G3PSR are similar to those used in MGGP with two main differences: the offspring should be admissible, as defined by the grammar, and in choosing the model terms that undergo crossover or mutation. Once an individual is selected from the population using tournament selection, the variation step is biased in the same manner as in [56] by setting the probability of choosing a model term from the individual proportional to its coefficient. The coefficients are first normalized using (4) and then the probability is calculated using (5), resulting in a higher chance of choosing model terms that have low or zero coefficients.

(4) $$\bar{\theta_{i}}=\frac{\sum_{i=1}^{q}\left|\theta_{i}\right|-\left|\theta_{i}\right|}{\sum_{i=1}^{q}\left|\theta_{i}\right|}$$

(5) $$P=\frac{exp\bar{\theta_{i}}}{\sum_{i=1}^{q}exp{\bar{\theta }}_{i}}$$

Grammars have been used in genetic programming to restrict the search space to increase interpretability and increase accuracy by incorporating prior knowledge about the problem. Several variants of grammar-based genetic programming, such as context-free grammar genetic programming [57] and grammatical evolution [51], have been applied successfully including the identification of nonparametric models of mechanical systems [58]. Context-free grammar is a four-tuple (N,Σ,P,S) used in computer science to generate syntactically correct sentences, where S is the start symbol, Σ is the set of terminal symbols, N is the set of all nonterminal symbols, and P is the set of production rules. The configuration of the G3PSR hyperparameters is given in Table 2, and the production rules are given in Table 3.

### 2.3. TS Fuzzy Model

TS fuzzy model [59,60] used in comparative study is defined as a set of *R* if-then rules with locally linear models in rule consequents:

(6) $${\hat{m}}_{i}=p_{i1}x+p_{i2}\varepsilon +p_{i3}$$

where *i* = 1, 2, …, *R*, $p_{i1}$, $p_{i2}$ and $p_{i2}$ are the rule-consequent parameters estimated using the least square method. The model output is calculated through interpolations of linear models between centroids of fuzzy clusters in the input space corresponding to rule antecedents:

(7) $$\hat{m}=\frac{\sum_{i=1}^{R}w_{i}{\hat{m}}_{i}}{\sum_{i=1}^{R}w_{i}}$$

where $w_{i}$ is a weight of *i*-th rule calculated as a product of Gaussian membership functions of input variables *x* (trolley position) and *ε* (strain)

(8) $$w_{i}=exp\left(-\frac{{\left(x-x_{i}\right)}^{2}}{2\sigma_{xi}^{2}}\right)exp\left(-\frac{{\left(\varepsilon -\varepsilon_{i}\right)}^{2}}{2\sigma_{\varepsilon i}^{2}}\right)$$

where $x_{i}$, $\varepsilon_{i}$, $\sigma_{xi}$, and $\sigma_{\varepsilon i}$ are the rule antecedent parameters corresponding to the expected values ($x_{i}$ and $\varepsilon_{i}$ coordinates of fuzzy clusters centroids in the input Cartesian space) and their standard deviations ($\sigma_{xi}$ and $\sigma_{\varepsilon i}$).

The TSF model identification was performed using a combination of the fuzzy subtractive clustering algorithm [61] and the least square method. The subtractive algorithm was applied to derive the *R* rule’s antecedents from measurement data through an iterative procedure that starts with the calculation of the potential of each *k*-th data point to be a cluster center based on a squared distance to the other data points:

(9) $$P\left(d_{k}\right)=\sum_{j=1}^{n}exp\left(-\frac{{∥d_{k}-d_{j}∥}^{2}}{{\left(\frac{r_{a}}{2}\right)}^{2}}\right)$$

where $r_{a}$ is a positive constant called cluster radius. The data point with the highest potential is chosen as the first cluster center. In each next *i*-th step, the potential of all remaining data points is reduced according to their distance to the cluster center $c_{i-1}$ selected in the previous step:

(10) $$P\left(d_{k}\right)=P\left(d_{k}\right)-P\left(c_{i-1}\right)exp\left(-\frac{{∥d_{k}-c_{i-1}∥}^{2}}{{\left(\frac{r_{b}}{2}\right)}^{2}}\right)\;$$

where $\;r_{b}=br_{a}$, and *b* is a positive parameter called the squash factor. The termination condition is defined twofold [61]. The upper and lower thresholds of data points’ potential are determined using $\xi_{1}$ and $\xi_{2}$ called the accept and reject ratio, respectively,

(11) $$\xi_{1}P_{1}<P_{i}<\xi_{2}P_{1}$$

where *P*_1_ is the potential of the data point chosen as the first cluster center. Thus, the cluster centroid is either accepted if $\;P_{i}>\xi_{1}P_{1}$, or rejected if $P_{i}<\xi_{2}P_{1}$, and the algorithm is terminated. If condition (11) is satisfied, the shortest distance $d_{min}$ between $d_{k}$ and all previously found centroids is verified using condition (12). The data point is accepted as the centroid if condition (12) is satisfied, otherwise, the data point with the next highest potential is tested, and the algorithm is terminated if all data points violate condition (12).

(12) $$\frac{d_{min}}{r_{a}}+\frac{P_{i}}{P_{1}}\geq 1$$

## 3. Results of Identification Experiments

**Figure 5:**
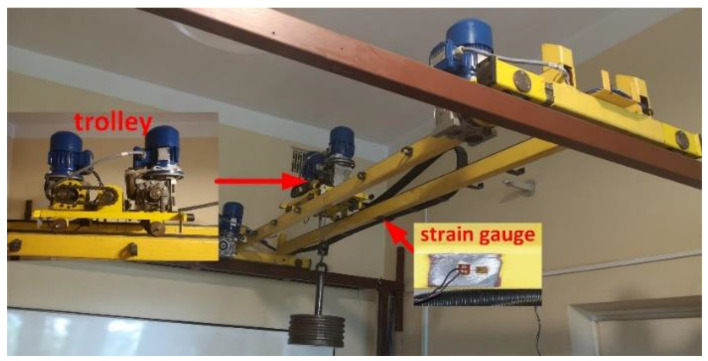
The laboratory stand.

The data used in the identification procedure were obtained from experiments carried out on a double girder overhead crane installed in a laboratory (Figure 5). The trolley is driven by an AC gear motor with a gear ratio of 15.5, operating at 1400 rpm and output power of 0.12 kW. The girders’ length, trolley wheelbase, and traveling range are 2.4 m, 0.3 m, and 1.8 m, respectively. The trolley position *x* along the girders is measured using the incremental encoder installed on the trolley’s wheel with a resolution of 200 pulses per revolution (ppr). The strain gauge supplied by the ADAM 3016 input module is applied to measure the strain at the middle point of the girder. The data from sensors are sampled at 10 Hz using a PC with 16GB RAM and Quad Core 4GHz Intel Core i7-6700K CPU running Windows 10 and MATLAB R2020.

**Figure 6:**
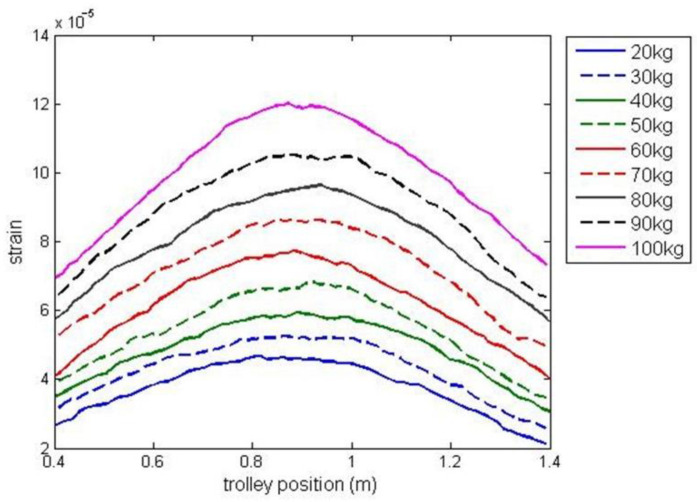
Experimental data partition into training and validation data (solid line), and testing data (dashed line).

A series of experiments with different payload masses within the range of 20 kg and 100 kg were carried out to collect training and testing data for identification. It was assumed, that payload weight estimation is performed based on the signal from the strain gauge located at the midpoint of the girder and the trolley position ranging in *x* = [0.4 m, 1.4 m] (with the strain gauge sensor at the midpoint of this interval). Figure 6 presents the experimental data obtained in experiments in which the trolley transported payload along the girder. The experimental data used to identify G3PSR and MGGP models were partitioned into three sets, the training set, validation set, and testing set. The training set is used to minimize (2) for G3PSR and (1) for MGGP, and the root mean square error (RMSE) (13) on the training set is used as the fitness function in G3PSR and MGGP. The validation set is used for model selection, i.e., the model with the lowest RMSE on the validation set is stored as the best model, while the testing set is used to evaluate the performance of the obtained model. The testing set comprises data from experiments with payload masses of 30, 50, 70, and 90 kg (Figure 6), while the rest of the experimental data (payload mass: 20, 40, 60, 80, and 100 kg) are combined and split randomly with a 70:30 ratio into the training and validation sets, respectively. The performances of the models were compared using relative error (RE), mean relative error (MRE), and RMSE (13), where m and $\hat{m}$ are the real and model-estimated values of weight, respectively, and n is the number of sample data.

(13) $$RE=\frac{\left|m-\hat{m}\right|}{m},\;\;MRE=\frac{1}{N}\sum_{i=1}^{n}\frac{\left|m-\hat{m}\right|}{m},\;\;RMSE=\sqrt{\frac{\sum_{i=1}^{n}{\left(m_{i}-{\hat{m}}_{i}\right)}^{2}}{n}}$$

**Figure 7:**
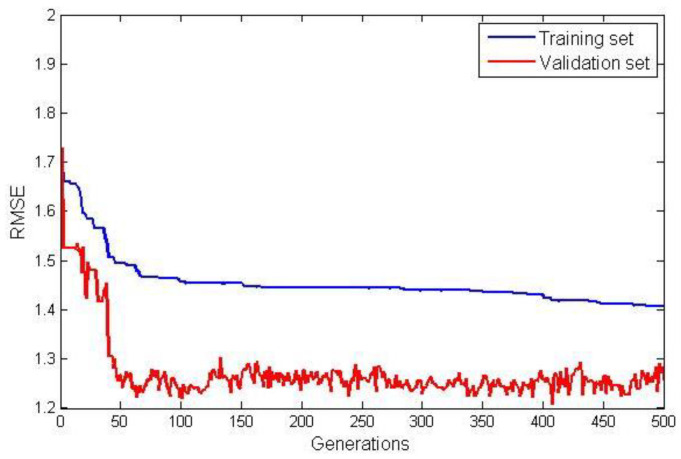
RMSE for training and validation data for the G3PSR.

**Figure 8:**
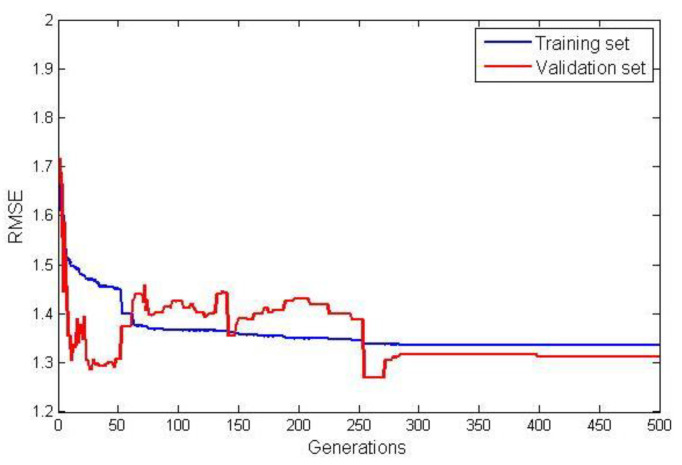
RMSE for training and validation data for the MGGP.

The model terms and their corresponding coefficients for G3PSR and MGGP static models obtained in identification experiments are given in Table 4. The value of the fitness function (RMSE) for both the training and validation data sets are shown in Figure 7 and Figure 8, for the G3PSR and MGGP models, respectively. Since both strategies use the performance on the validation set as the criteria for selection, the final models had RMSEs on the validation set of 1.2077 and 1.2565 by applying the G3PSR and MGGP techniques, respectively.

In the case of the TSF model identified using the combination of subtractive clustering and least square methods, the best model was obtained by setting by trial and error the cluster radius and squash factor to $r_{a}=0.7$ and *b* = 1.1, respectively. For this setting, a clustering technique partitioned input space into eight clusters generating rules with premise and consequent parameters given in Table 5.

The comparison of model performances for testing data in terms of accuracy and complexity is presented in Table 6. The G3PSR and MGGP models show similar performances for testing data: RMSE and MRE are 1.7813 and 0.0285 for G3PSR, while for MGGP they are 1.8069 and 0.0283, respectively. The TSF model performs slightly worse, as the RMSE and MRE are 1.8875 and 0.0294, respectively. In terms of the number of parameters to be estimated, the G3PSR and MGGP models have 14 and 17 parameters, respectively, while the number of parameters of the TSF model (56 parameters) is significantly greater. The G3PSR model notably outperforms the other models in terms of computational complexity, since the execution time (mean value from $10^{3}$ runs plus/minus standard deviation) is $5.2\times 10^{-3}\pm 5.8\times 10^{-7}$ ms, while the MGGP and TSF models’ execution times are $80.4\times 10^{-3}\pm 5.5\times 10^{-3}$ ms and $367.5\times 10^{-3}\pm 7.6\times 10^{-3}$ ms, respectively.

**Figure 9:**
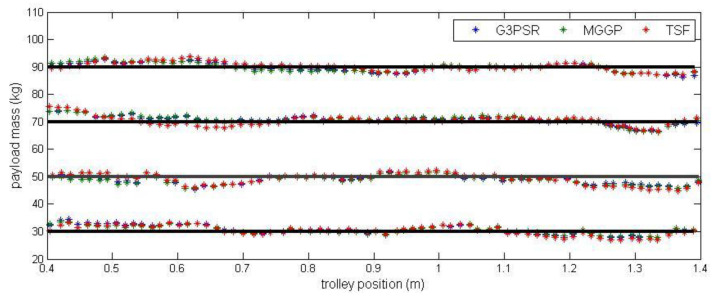
Payload estimated mass along the test trajectories.

**Figure 10:**
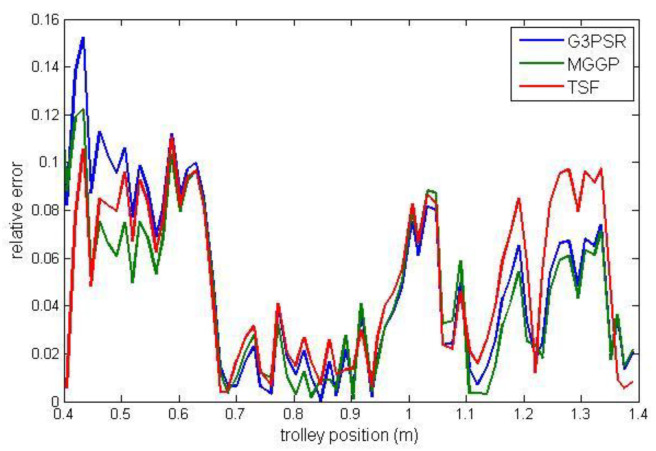
Relative error for estimated mass 30 kg.

**Figure 11:**
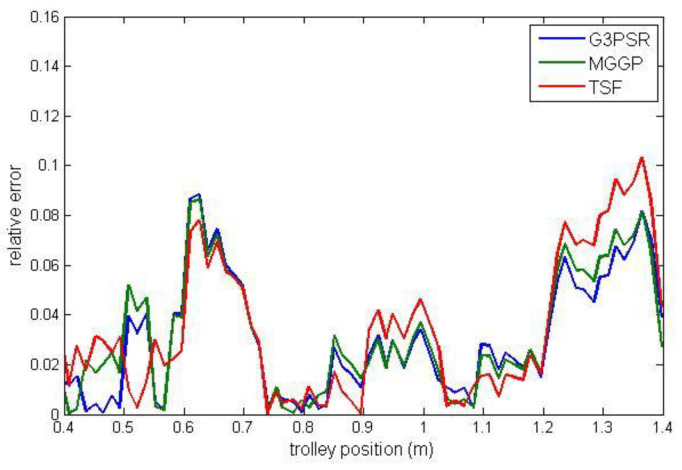
Relative error for estimated mass 50 kg.

**Figure 12:**
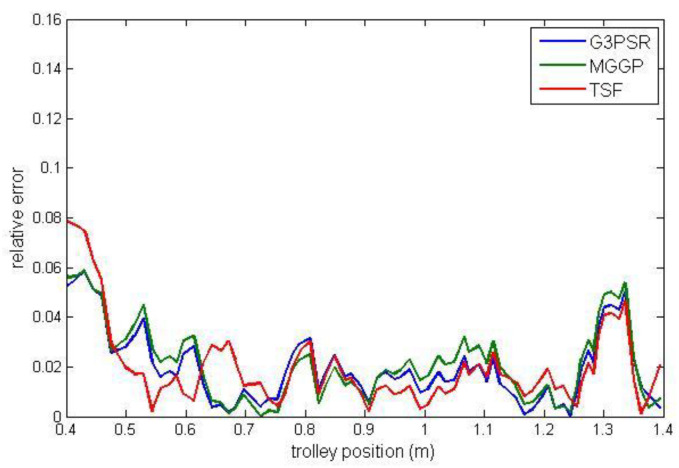
Relative error for estimated mass 70 kg.

**Figure 13:**
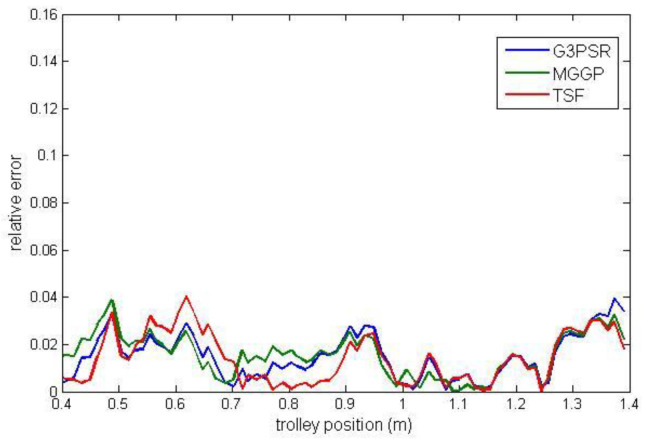
Relative error for estimated mass 90 kg.

Payload estimated mass along the test trajectories is compared in Figure 9, while the relative errors between the real and model-estimated values of weight for G3PSR, MGGP, and TSF models estimating mass 30 kg, 50 kg, 70 kg, and 90 kg are presented in Figure 10, Figure 11, Figure 12 and Figure 13, as well as in Table 7 where the models’ performances are compared using the MRE and maximum value of RE. Obviously, the accuracy in weight estimation increases with the increase in the payload mass. The models’ performances are similar for a payload mass of 90 kg. The maximum relative error is 0.0395, 0.0388, and 0.0402 for G3PSR, MGGP, and TSF models, respectively. The second obvious conclusion, which comes from Figure 9, Figure 10, Figure 11, Figure 12 and Figure 13 is that dividing a girder length into more sections with strain gauges at midpoints enhances estimation accuracy at the cost of more sensors and models to be used. It should be also noticed that the models were developed for the payload weight range of 20-100 kg, and we can expect that model complexity can rise with the increase in the range of the payload mass to be estimated. Thus, model optimization is necessary to save computational consumption.

**Figure 14:**
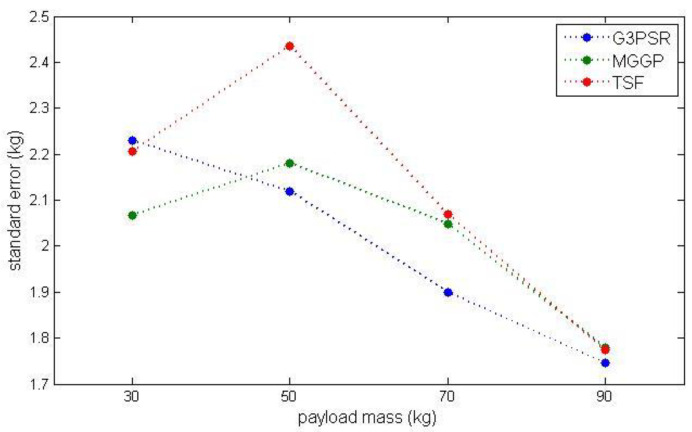
Standard error at testing points.

Uncertainty analysis is performed taking into account the effect of the strain signal variation. Another set of testing data obtained from eight experiments carried out on the laboratory stand was used to determine the standard deviation of strain signal with respect to the data used to identify the models assumed as the nominal data for comparison. The nominal strain input data used for models identification were then perturbed with a standard deviation of $\sigma =0.8463\times 10^{-6}$. The comparison of model performances at testing operating points for nominal input data  (ε) and strain signal deviated by ε±σ is presented in Table 8 using RMSE and MRE. All models exhibit a similar increase in RMSE (2.2115, 2.2104, and 2.2889 for G3PSR, MGGP, and TSF models, respectively) when the nominal trajectory of strain signal is deviated by ε+σ**,** while the G3PSR model is slightly less affected by strain signal perturbation ε−σ, since the RMSE is 2.0835, 2.2169, and 2.2169 for G3PSR, MGGP, and TSF models, respectively. Taking into account the nominal and deviated strain signal, the uncertainty of the models’ output (estimated payload weight) is expressed by standard error calculated according to (14), and the results are presented in Table 9 and Figure 14 (where the dotted line is a linear interpolation between testing points). The general tendency observed in Figure 14 and Table 9 is that the standard error decreases with the increase in the payload weight from 30 to 90 kg, from 2.2297 to 1.7460 for the G3PSR model, and from 2.0670 to 1.7786 and from 2.2066 to 1.7740 for MGGP and TSF models, with the exception of 50 kg.

(14) $$s=\sqrt{\frac{\sum_{i=1}^{N}{\left(m_{i}-{\hat{m}}_{i}\right)}^{2}}{N-1}}$$

## 4. Conclusions

The paper compares the data-driven soft-computing models for payload weight estimation for an overhead crane. The payload mass is estimated based on a local strain of a crane’s girder and the trolley position. Two genetic programming variants are used for model optimization, multi-gene genetic programming, a novel technique and grammar-guided genetic programming with sparse regression. The genetic programming methods are compared with the TSF model evolved using the fuzzy subtractive clustering and least square methods. The comparative study is presented in terms of accuracy and complexity. The G3PSR and MGGP models show similar estimation accuracy with an RMSE of 1.7813 and 1.8069 respectively, and slightly better than in the case of the TSF model with an RMSE of 1.8875. The relative error decreased for all models identified as the payload mass increased, this is due to the fact that the absolute error does not differ by a significant amount depending on the payload mass while the relative error is normalized by the payload mass, which, when increased, results in a decrease in the relative error. The model complexity expressed as the number of model parameters to be estimated is lowest for the G3PSR model (14 parameters) in comparison to the MGG and TSF models at 17 and 56 parameters, respectively. The significant difference we observe in the model execution time is that the G3PSR outperforms the other models with a mean execution time of $5.2\times 10^{-3}$ ms, while the MGGP model and the TSF model had mean execution times of $80.4\times 10^{-3}$ ms and $367.5\times 10^{-3}$ ms, respectively. The direct implementation of the input–output function decreased the computational time by approximately 98.6% and 78.1% for the G3PSR and MGGP models, respectively, when compared to the TSF model. Future works will be focused on testing the proposed approach on a crane system with a larger girder length and payload weight range. The G3PSR algorithm would also be applied to the inverse modeling approach to estimate the strain/stress of a girder [18] for a given payload mass, as well as in other static and dynamic model optimization problems [62,63].

## Figures and Tables

**Table 1:** Configuration of the MGGP hyperparameters.

Parameters	Settings
Function set	×, √, *inv*
Terminal set	*x*, *ε*
Population size	100
Number of generations	500
Initialization	Ramped Half-and-Half
Maximum number of genes	25
Maximum tree depth	5
Tournament size	2
Crossover probability	0.84
Mutation probability	0.14
Direct reproduction	0.02

**Table 2:** Configuration of the G3PSR hyperparameters.

Parameters	Settings
Set of nonterminal symbols *N*	×, √, *inv*
Set of terminal symbols Σ	*x*, *ε*
Population size	100
Number of generations	500
Initialization	Probability tree creation 2 (PTC2)
Number of candidate model terms	25
Maximum tree depth during initialization	8
Tournament size	2
Subtree crossover probability	0.75
High-level crossover probability	0.15
Mutation probability	0.1
Sparsification parameter *λ*	0.001

**Table 3:** Grammar used to obtain the G3PSR model.

〈S〉	::=	〈exp〉
〈exp〉	::=	〈op_b_〉 〈exp〉 〈exp〉 | 〈op_u_〉 〈exp〉 | 〈T〉
〈op_b_〉	::=	×
〈op_u_〉	::=	√ | *inv*
〈T〉	::=	*x* | *ε*

**Table 4:** The model terms and coefficients generated by G3PSR and MGGP.

G3PSR		MGGP	
Model Coefficients	Model Terms	Model Coefficients	Model Terms
−0.0058	$\frac{1}{\varepsilon }$	−5.2140 × 10^5^	1
4.5510 × 10^22^	$\frac{x^{5}\varepsilon^{6}}{\sqrt{\varepsilon }}$	879.6109	$x\varepsilon^{2}$
15.6505	$\frac{1}{\sqrt[{4}]{\varepsilon }}$	−1.2297 × 10^4^	$x\sqrt[{4}]{\varepsilon }$
1.1976 × 10^5^	$x^{3}\varepsilon$	−5.0552 × 10^5^	$\sqrt{x}$
1.6163 × 10^−7^	$\frac{\sqrt[{4}]{\varepsilon }}{\varepsilon^{2}x\sqrt{x}}$	8.3531 × 10^4^	x
−161.6078	x	8.2332 × 10^5^	$\frac{\varepsilon }{x}$
4.4107 × 10^−4^	$\frac{x\varepsilon }{\sqrt{x}}$	2.0059 × 10^4^	$\frac{\sqrt{\varepsilon }}{x}$
18.2220	$x^{2}$	1.5669 × 10^4^	$\sqrt{x}\sqrt[{4}]{\varepsilon }$
−3.2326 × 10^13^	$\varepsilon^{3}x$	−1.3150 × 10^5^	$x\sqrt{\varepsilon }$
2.3790 × 10^−8^	$\frac{x}{\varepsilon^{2}}$	3.3940 × 10^5^	$\sqrt{x}\sqrt{\varepsilon }$
14.1349	$\frac{x^{4}}{\sqrt[{8}]{\varepsilon x}}$	−3.0590 × 10^5^	$\sqrt{\varepsilon }$
−3.2074	$x^{8}\sqrt{x}$	1.2318 × 10^5^	$x^{2}\sqrt{\varepsilon }\sqrt[{4}]{\varepsilon }$
9.7761 × 10^7^	$\sqrt{\varepsilon^{3}}$	9.8117 × 10^3^	$\frac{\sqrt[{4}]{\varepsilon }}{\sqrt{x}}$
−5.7385 × 10^−9^	$\frac{1}{\varepsilon^{2}x\sqrt[{8}]{x^{5}}}$	1.1880 × 10^5^	$\frac{1}{\sqrt[{4}]{x}}$
		8.2782 × 10^5^	$\sqrt[{4}]{x}$
		−3.7918 × 10^3^	$x^{2}$
		−982.1465	$\frac{\sqrt[{4}]{\varepsilon }}{x^{2}}$

**Table 5:** TSF model parameters.

Rule Number	Antecedent (Gaussian) Parameters		Consequent (Linear Function) Parameters
*i*	$\left[\sigma_{xi},\;x_{i}\right]$	$\left[\sigma_{\varepsilon i},\;\varepsilon_{i}\right]\times 10^{-5}$	$\left[p_{1i},\;p_{2i},\;p_{3i}\right]$
1	[0.250, 0.8652]	[2.447, 5.8133]	$\left[-60.65,\;3.97\times 10^{3},\;1.288\times 10^{2}\right]$
2	[0.250, 0.8523]	[2.447, 9.4161]	$\left[-286.01,\;5.605\times 10^{5},\;286.19\right]$
3	[0.250, 1.2646]	[2.447, 5.2689]	$\left[32.11,-1.4716\times 10^{6},\;228.17\right]$
4	[0.250, 0.4850]	[2.447, 5.0614]	$\left[5.81\times 10^{3},-2.467\times 10^{8},\;1.824\times 10^{4}\right]$
5	[0.250, 1.2048]	[2.447, 3.3429]	$\left[119.45,-1.365\times 10^{5},-270.93\right]$
6	[0.250, 1.2379]	[2.447, 9.1502]	$\left[2.16,\;1.211\times 10^{6},\;34.67\right]$
7	[0.250, 0.5311]	[2.447, 3.4812]	$\left[3.99\times 10^{3},-1.309\times 10^{8},-8.82\times 10^{3}\right]$
8	[0.250, 0.4473]	[2.447, 7.4674]	$\left[-230.99,\;3.9165\times 10^{7},\;5.935\times 10^{3}\right]$

**Table 6:** Comparison of models complexity and performances for testing data.

	G3PSR	MGGP	TSF
RMSE	1.7813	1.8069	1.8875
MRE	0.0285	0.0283	0.0294
No. of parameters	14	17	56
Mean execution time (ms) ± standard deviation	$5.2\times 10^{-3}$ $\pm 5.8\times 10^{-7}$	$80.4\times 10^{-3}\pm 5.5\times 10^{-3}$	$367.5\times 10^{-3}\pm 7.6\times 10^{-3}$

**Table 7:** Models performances at testing operating points.

	G3PSR		MGGP		TSF	
Payload Mass (kg)	MRE	max RE	MRE	max RE	MRE	max RE
30	0.0502	0.1523	0.0449	0.1570	0.0499	0.1108
50	0.0302	0.0883	0.0320	0.0862	0.0344	0.1033
70	0.0200	0.0585	0.0219	0.0606	0.0207	0.0802
90	0.0148	0.0395	0.0156	0.0388	0.0142	0.0402

**Table 8:** Models performances at testing operating points for deviated strain input signal.

	G3PSR			MGGP			TSF		
	*ε*	*ε* + *σ*	*ε* − *σ*	*ε*	*ε* + *σ*	*ε* − *σ*	*ε*	*ε* + *σ*	*ε* − *σ*
RMSE	1.7813	2.2115	2.0835	1.8069	2.2104	2.2169	1.8875	2.2889	2.2169
Payload mass (kg)				MRE					
	ε	ε+σ	ε−σ	ε	ε+σ	ε−σ	ε	ε+σ	ε−σ
30	0.0502	0.0793	0.0504	0.0449	0.0748	0.0469	0.0499	0.0771	0.0532
50	0.0302	0.0255	0.0487	0.0320	0.0246	0.0518	0.0344	0.0348	0.0474
70	0.0200	0.0296	0.0155	0.0219	0.0318	0.0172	0.0207	0.260	0.0196
90	0.0148	0.0161	0.0169	0.0156	0.0172	0.0166	0.0142	0.0177	0.0153

**Table 9:** Standard error at testing operating points.

	G3PSR	MGGP	TSF
Payload Mass (kg)	Standard Error (kg)		
30	2.2297	2.0670	2.2066
50	2.1200	2.1813	2.4352
70	1.9005	2.0492	2.0698
90	1.7460	1.7786	1.7740

## Data Availability

The data presented in this study are available on request from the corresponding author.

## Appendix A. Symbols and Definitions

This appendix consists of a list of all the symbols used in this paper along with their definitions (Table A1) in addition to abbreviations (Table A2).

**Table A1:** List of symbols and their definitions.

Variable	Definition
b, c, d	Squash factor, cluster center, distance between cluster centers
m	Suspended payload mass
$p_{i1},\;p_{i2},\;p_{i3}$	Rule consequent parameters
$r_{a}$	Cluster radius
$w_{i}$	Weights of *i*-th rule
x	Trolley position
N	Set of all nonterminal symbols
P	Set of production rules (also potential of chosen datapoint as cluster center and probability of selection)
S	Start symbol
ε	Strain
$\zeta_{1},\;\zeta_{2}$	Accept ratio, reject ratio
η	Step size
θ	Model term coefficients
$\bar{\theta }$	Normalized coefficients
λ	Sparsification parameter
ϕ	Regressor matrix
Σ	Set of all terminal symbols

**Table A2:** List of abbreviations and their definitions.

Abbreviation	Definition
GP	Genetic programming
G3PSR	Grammar guided genetic programming with sparse regression
mAPG	Monotone accelerated proximal gradient descent
MGGP	Multi-gene genetic programming
PTC2	Probability tree creation 2
TSF	Takagi–Sugeno fuzzy
